# Supplementary material for: Identification of Ku70 Domain-Specific Interactors Using BioID2
Source: Cells. 2021 Mar 14;10(3):646. doi: 10.3390/cells10030646 (PMC8001828; doi:10.3390/cells10030646)
Supplement: Supplementary file 1 [file cells-10-00646-s001.zip › Supplemental Figures.pdf]

BioID2 Cloning	Primer Name	Sequence (5'-3')
Wild-type Ku70	Ku70 HpaI Fwd	ATCGTGGTTAACCGGATGTCAGGGTGGGAGTCATATTACAAAACCGAG
	Ku70 BamHI Rev	TACGATGGATCCCGCGCAGTCCTGGAAGTGCTTGGTGAGGGC
Ku70 NLS	NLS-BamHI Fwd	GATCC**ATG**AAAGTTACCAAGAGAAAACACGATAATGAAGGTTCTGGAAGCAAAAGGCCCAAGG
	NLS-BamHI Rev	GATCCCTTGGGCCTTTTGCTTCCAGAACCTTCATTATCGTGTTTTCTCTTGGTAACTTT**CAT**G
Ku70 vWA into NLS-BioID2	vWA NheI Fwd	AAGCTCGCTAGCATGTCAGGGTGGGAGTCATATTACAAAACCGAGGGCG
	vWA HpaI Rev	TACGACGTTAACCTCCTTGGCGCGAACCTTCCGCAACAGG
Ku70 ΔvWA	ΔvWA NheI Fwd	AAGCTCGCTAGC**ATG**ACCAGGAAGCGAGCACTCAGCAGGTTAAAG
	ΔvWA BamHI Rev	TACGACGGATCCTGAGTCCTGGAAGTGCTTGGTGAGGGC

Figure S1. Forward (fwd) and reverse (rev) primers used to clone Ku70 fragments into the BioID2 (MCS-BioID2-HA) plasmid. Bolded letters indicate novel start codons added to the construct. Underlined letters represent the indicated restriction enzyme recognition and cut site.

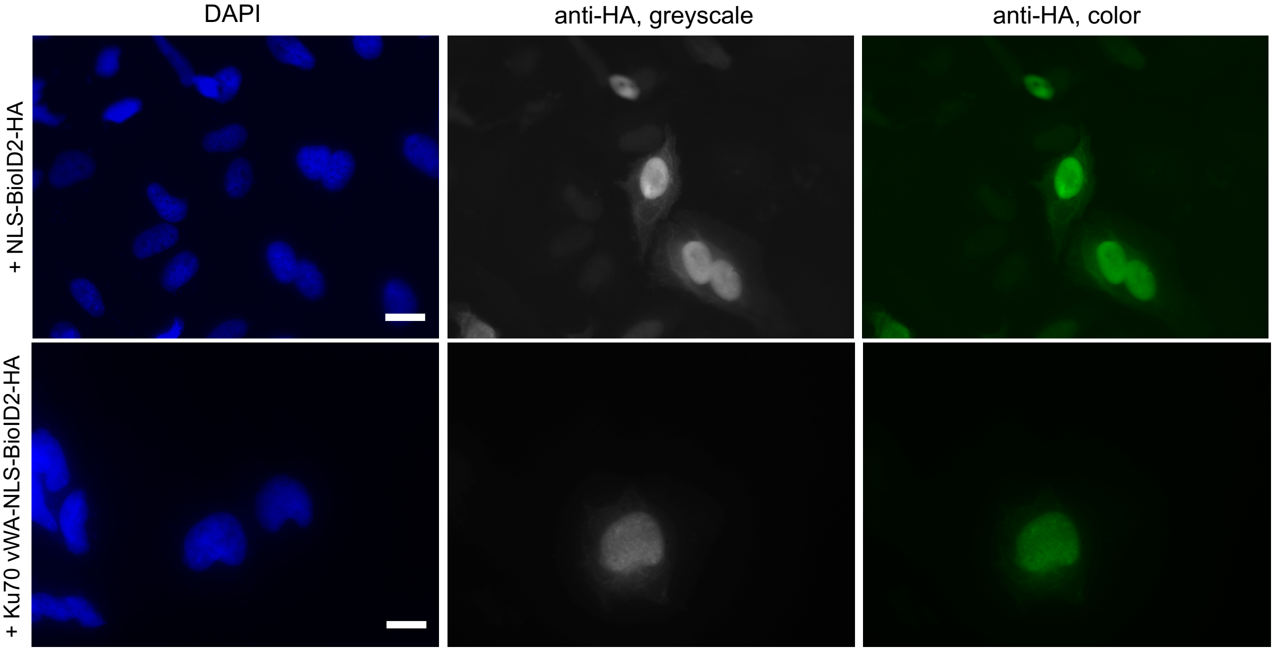

Figure S2. Testing the nuclear localization and targeting capacity of the Ku70 nuclear localization signal (NLS). Indirect immunofluorescence of HeLa cells transiently transfected with NLS-BirA*-HA and Ku70 vWA-NLS-BirA*-HA (BioID2 fusion constructs) using mouse anti-HA antibody (Sigma). Images were taken at 40X magnification and white bar denotes 20 μm.

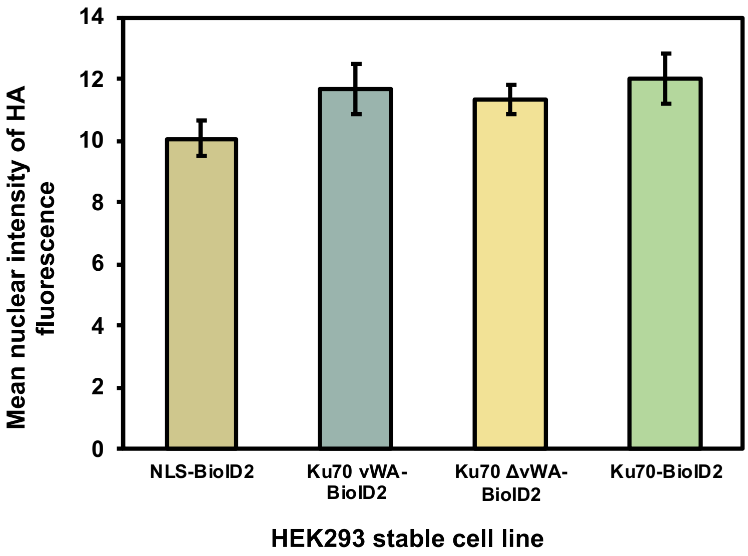

Figure S3. Mean nuclear intensities for HA immunofluorescence images of each BioID2 construct depicted in Figure 2c. Regions of Interest (ROI), area, and mean pixel intensities (including the minimum and maximum intensity for each ROI, ranging between 0 and 255) were acquired using the ImageJ program. Nucleus-specific ROI were determined by overlaying the binary DAPI image outline mask onto the HA fluorescence image and measuring the mean nuclear intensity for the ROIs before averaging all mean intensities and subtracting the HEK293 negative control for each of the four stable cell lines. Mean nuclear intensity of HA fluorescence was plotted with standard error of the mean (± SEM) indicated.

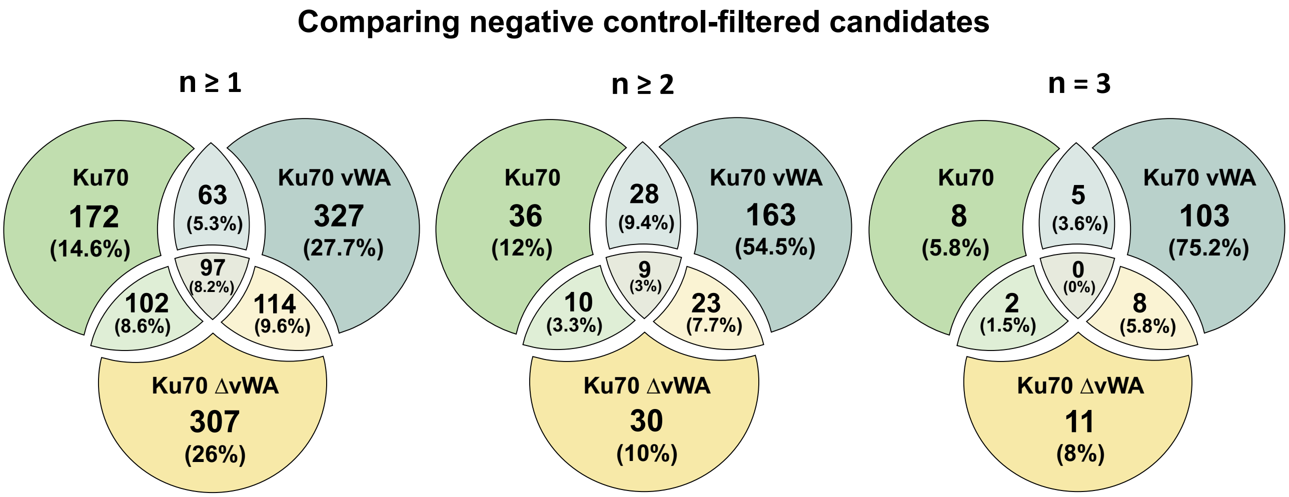

Figure S4. Alternative comparisons of BioID2 results using Ku70 segmented constructs. Ku70-, Ku70 vWA-, and Ku70 ΔvWA-BioID2 candidates were filtered by subtracting negative control proteins identified in two or more (n ≥ 2) biological MS replicates of either the HEK293 cell line or the NLS-BioID2 cell line. Additionally, all keratin proteins were removed. Candidates from each construct are compared here using different criteria were candidates that appeared in one or more (n ≥ 1), two or more (n ≥ 2), or three (n = 3) biological replicates are compared.

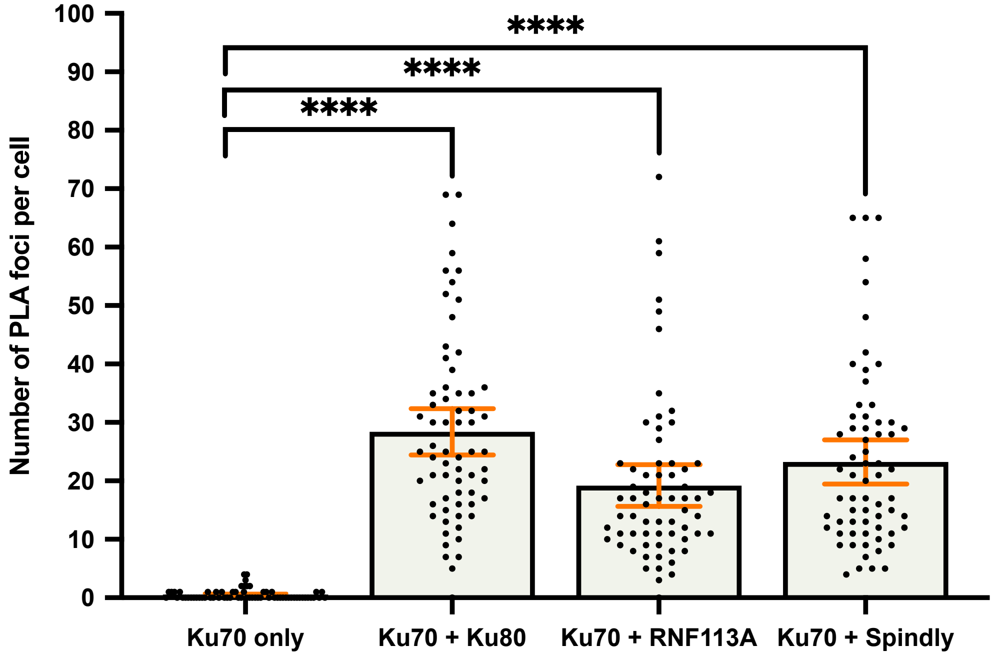

Figure S5. Quantification of the number of PLA foci per cell. PLA foci were counted using ImageJ. Bar graphs indicate mean number of nuclear PLA foci and dots indicate exact number of nuclear foci counted for each cell (n = 65 cells from at least 2 separate PLA experiments for each PLA condition) with 95% confidence intervals depicted in orange. The negative control (Ku70 only) mean was compared to Ku70 with Ku80 ($p = 2.50 \times 10^{-21}$), RNF113A ($p = 1.53 \times 10^{-15}$), or Spindly ($p = 3.97 \times 10^{-18}$) using unpaired, two-tailed t-tests assuming unequal variance. **** indicates that $p < 0.0001$.

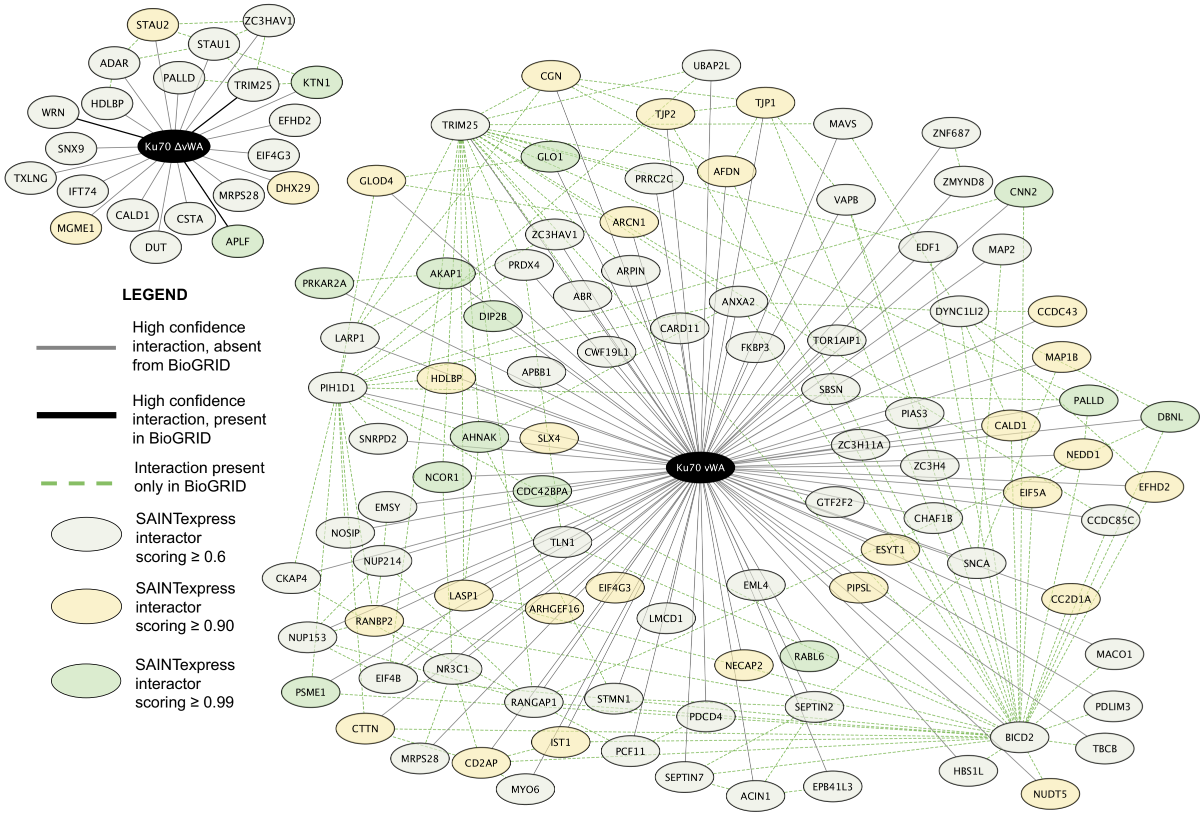

Figure S6. Protein network of high confidence SAINTexpress candidate interactors (including those with a SAINTexpress score of ≥0.6) for Ku70 ΔvWA- and Ku70 vWA BioID2 analysis. The SAINTexpress score for each candidate is indicated by the node colour. If there was any previous evidence of an interaction between two candidates on the BioGRID database, this was also indicated by the style and colour of the edges (lines).
